# A Case of Post-Traumatic Retrograde and Anterograde Loss of Autobiographical Memory in the Absence of Medial Temporal Lobe Lesion

**DOI:** 10.7759/cureus.11898

**Published:** 2020-12-04

**Authors:** Ravindi Gunasekara, Shahzad Chida, Minahil Shahid, Gulshan Begum, Ayodeji Jolayemi

**Affiliations:** 1 Psychiatry, Medical University of the Americas, Charlestown, KNA; 2 Psychiatry, Interfaith Medical Center, Brooklyn, USA; 3 Psychiatry, American University of Antigua College of Medicine, Coolidge, ATG

**Keywords:** anterograde amnesia, retrograde amnesia, medial temporal lobe, autobiographical memory, case report, post traumatic memory loss, henry molaison, temporal lobe, procedural memory, memory formation

## Abstract

We present an unusual case of a middle-aged patient with a near-total loss of autobiographical memory; the memory of one's own personal history and personal identity following a motor vehicle accident. The nature of his autobiographical memory loss was anterograde and retrograde amnesia, with the preservation of procedural learning, including an extensive set of medical skills, which he attributed to his career as a psychiatrist. Of significance was the absence of any traumatic changes in the medial temporal lobe structures, hippocampal formation, amygdala, and entorhinal cortex on brain imaging. The significance of our findings is discussed in the context of the evolving theories of the role of medial temporal lobe structures in memory formation.

## Introduction

The famous case of Henry Molaison (H.M.) has been pivotal in understanding the human brain and has provided an important scientific framework for the organization and distribution of memory functions, as well as theories involving memory consolidation, storage, and retrieval [1]. H.M underwent a bilateral medial temporal lobe resection including hippocampal formation, amygdala, and entorhinal cortex, for treatment-refractory seizures [2]. He exhibited complete loss of social memory of the day-to-day events following his operation, as well as partial retrograde amnesia, all in the absence of impairment of his personality or semantic knowledge [3]. He could no longer recognize the hospital staff nor could he find his way to the bathroom. He had no recollection of the daily events of his hospital life. He also could not remember the death of his favorite uncle three years prior, nor did he recall trivial events that had occurred prior to his admission to the hospital [3]. However, he was still able to form procedural memories such as learning new motor skills, and despite significant impairment on spatial memory tests, he was able to construct a cognitive map of the spatial layout of his house after daily locomotion from room to room [4]. 

The case of H.M. helped establish the theory that memory may be a distinctly temporal lobe function [1]. After H.M., there have been many subsequent studies with similar patterns of memory loss in individuals with radical resections of the brain [3]. Autobiographical memory is a form of declarative or explicit memory that refers to the storage of memories of events that have happened to a person. It involves a complex interplay of episodic memories of past events, self‐definition, emotion, visual imagery, executive function, and semantic processes including a recollection of facts and general knowledge. In a study by Noulhiane et al. [5], it was found that autobiographical memory was impaired in patients with right and left medial temporal lobe resections when compared to normal controls across all time periods. There have, however, been studies calling into question the focus on medial temporal lobe structures in autobiographical memory formation. For instance, Kirwan et al. [6] found that autobiographical recollection was impaired in patients with medial temporal lobe damage when memories were drawn from the recent past, but fully intact when memories were drawn from the remote past. They suggested taking into consideration the structures outside of the medial temporal lobe as playing a role in remote autobiographical memory. In such cases, the loss of remote autobiographical memory may be observed in patients with intact medial temporal lobe structures.

We present a case report of anterograde and retrograde amnesia, specifically, loss of autobiographical memory with preserved procedural memory, following a motor vehicle accident. The memory loss consisted of both remote and recent autobiographical memory with loss of personal identity. Of significance was the absence of any traumatic changes in the medial temporal lobe structures, hippocampal formation, amygdala, and entorhinal cortex on clinical brain imaging. We discuss the significance of our findings in the context of the changing understanding of the role of medial temporal lobe structures in memory formation.

## Case presentation

We present the case of a 44-year-old right-handed male with no reported psychiatric history or medical history. He sustained trauma in a motor vehicle accident with a right hip injury, lumbar spinal injury, and collar bone fracture. There were no reports of head trauma or loss of consciousness resulting from the injury. He was managed in the hospital for the fractures he sustained and was then transferred to a rehabilitation center for further treatment of the limitations in his mobility. He completed treatment but was unable to be discharged to the community due to depressed mood, impaired cognition, and disorientation. He was therefore transferred to an inpatient psychiatry unit for further management.

The patient did not demonstrate symptoms of an acute depressive episode during his initial interview. He had a depressed mood related to his recent trauma but denied anhedonia, low energy level, poor concentration, feelings of worthlessness, guilt, or suicidal ideations. The patient demonstrated no signs or symptoms of psychosis. No delusions were elicited. He denied excessive worrying, feeling on edge or keyed up, muscle stiffness, and restlessness. He denied palpitations, sweating, trembling, shortness of breath, chest pain, nausea, dizziness, headaches, or paresthesia. He denied depersonalization, derealization, having lapses in memory, or periods of time in which he was unable to account.

A significant finding on psychiatric assessment was his inability to recognize his face when looking into the mirror. He indicated he could not recall his name. He was disoriented to place and time. He was unable to recollect any memory of any recent trauma. He scored 9 out of 30 on the Folstein Mini-Mental State Exam (MMSE) and 3 out of 30 on Montreal Cognitive Assessment (MOCA) demonstrating deficits in all areas, including visuospatial/executive, naming, short-term and long-term memory, language, and orientation. A comprehensive assessment of his developmental history from childhood to adulthood demonstrated that he had significant impairments of autobiographical memory, including both episodic and self-reference semantic memory. He was unable to remember any childhood memories or memories of life events that occurred during adulthood. He had no recollection of his family, friends, or significant others. He was unable to recollect recent living situations, recent activities, or any other positive or negative memories. The only memory that he was able to recall was that he was a psychiatrist. In addition, he demonstrated extensive knowledge of clinical psychiatry and psychopharmacology. He was also able to draw diagrams of the brain with accurate labeling of different regions of the brain. However, he was unable to provide any details of events that occurred during his job as a psychiatrist in the past.

Repeated evaluation of the patient demonstrated that he had trouble forming new memories, evidenced by his inability to remember the occurrence of new events, or the names and faces of his treatment team, including the treating psychiatrists, social workers, and nurses. The treatment team had to introduce themselves to him with each daily encounter. He would forget what he ate for breakfast when asked one hour later and would often forget the fact that he had breakfast in the first place. In other aspects of his mental status examination, he was a cooperative and courteous man who enjoyed teaching medical students and residents from his reported experiences and skills in the field of psychiatry. His thought process was linear, logical, and goal-directed, with no deficits in quality of associations. His neurological examination revealed no significant focal neurological deficits in the cranial nerves. He had 3/5 bilateral upper extremity strength with difficulty clenching both fists given the weakness in his hand muscles, and 4/5 bilateral lower extremity strength with recurrent spasms and hyperreflexia. All other motor examinations including cerebellar signs were unremarkable.

**Figure 1:**
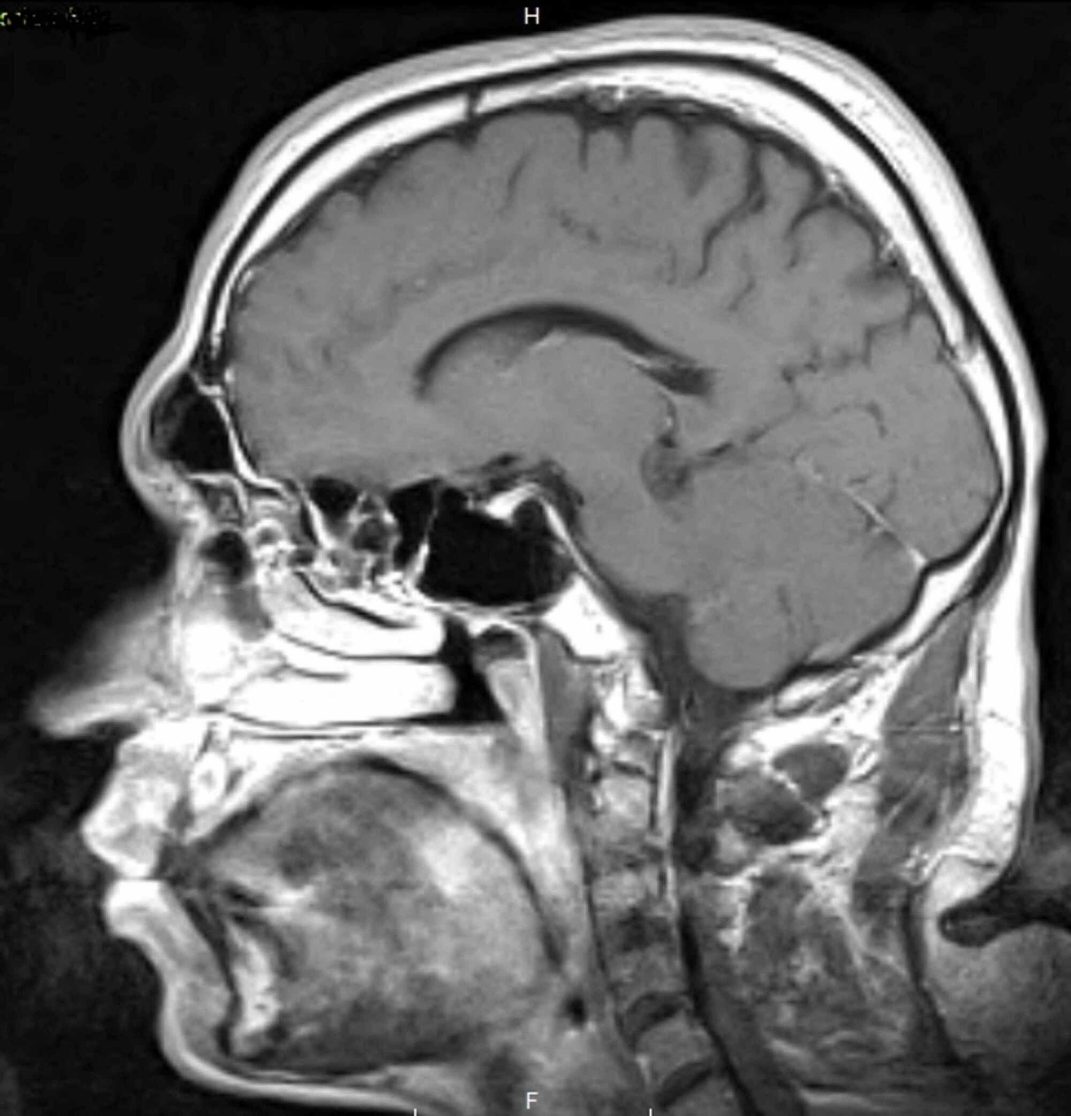
A sagittal plane MRI of the patient’s brain.

**Figure 2:**
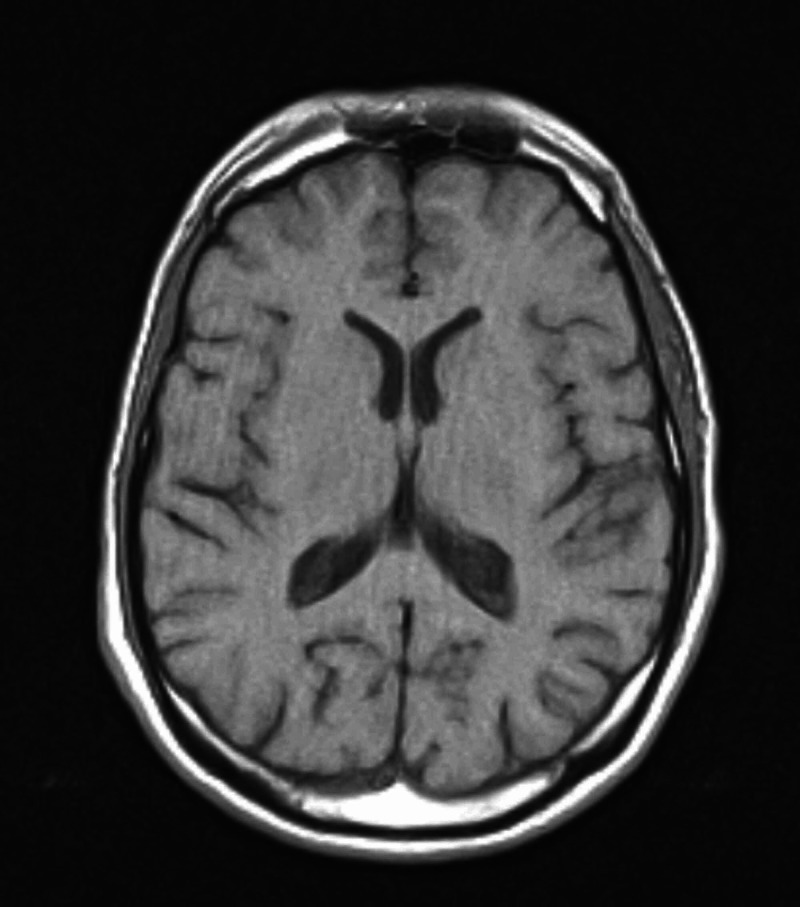
A transverse plane MRI of the patient’s brain.

Laboratory workup including comprehensive metabolic profile, complete blood count, hormonal workup, Vitamin B12, folate, and serology were all within normal limits. Brain imaging, including a CT and MRI, revealed no evidence of structural lesions. His medial temporal lobe, lateral temporal lobe, frontal lobe, parietal lobe, occipital lobe, and subcortical structures were all intact. No evidence of intracranial hemorrhage was observed, and there was no evidence of atrophy or ischemia (Figures 1 and 2).

A spinal CT revealed mild central canal narrowing at C5-6, non-specific changes in C9-10, mild degeneration which the radiologist indicated was appropriate for the patient’s age, and minimal malalignment in the mid-portion of the thoracic spine.

Over the course of the hospitalization, the patient underwent cognitive rehabilitation aimed at improving his memory impairment. However, there was no improvement in his remote and recent autobiographical memory during his hospital course. He maintained the same levels of memory impairment, only remembering a few pieces of information at different times related to his car accident and previous research topics. There remained an impairment in the recall of his identity and other episodic memory from both remote and recent times of his life. He continued to demonstrate intact procedural memory and continued to demonstrate skills that he reportedly learned as a medical professional. When attempts were made to see if he could improve on learning new events, he became amnesic for the recalled content shortly after a couple of hours. He was eventually discharged to an assisted care facility due to his continued impairment in memory and the need for additional supportive care.

## Discussion

This is a case of a patient presenting with anterograde and retrograde amnesia with preserved general semantic memory following a motor vehicle accident. He lost his personal identity in addition to autobiographical memory. He retained his semantic knowledge including extensive medical knowledge. Interestingly, the pattern of his memory loss closely resembled that of the famous case of H.M., who underwent bilateral medial temporal lobe resection and had a complete loss of memory of events following his surgery and partial retrograde amnesia in the absence of changes in personality or semantic knowledge. The patient's blood tests and CT/MRI studies were unremarkable, with a notable absence of traumatic changes in the medial temporal lobes.

Diagnosing and managing an unidentified individual with profound amnesia without any collateral information is a challenge. The differential diagnosis includes cognitive disorder, post-traumatic stress disorder (PTSD), dissociative disorders, traumatic brain injury, and factitious disorder/malingering. Although our patient shows significant impairment of cognition on the MMSE and MOCA, he does not meet the full criteria for mild cognitive dysfunction or dementia. PTSD is a possible diagnosis, although we do not know the precise history of trauma aside from the motor vehicle accident. He also did not display any other features of PTSD. A consideration, in this case, could also be, dissociative disorders, for example, dissociative fugue, depersonalization disorder, or dissociative identity disorder. Dissociative disorders are considered controversial diagnoses, for there are many disputes and professional skepticism over the meaning of the observed symptoms [7]. Patients with dissociative disorders possess extreme sensitivity to interpersonal trust and rejection [8]. In addition, the preservation of procedural memory and medical knowledge with the loss of his autobiographical memory is not typical of dissociative disorders. Given his report of being a psychiatrist and expressing knowledge in the field of psychiatry, one could consider that he might have been a patient who gained knowledge during the course of prior mental health contacts. He, however, did not demonstrate any symptoms of psychosis, mania, or depression acutely and he was not maintained on any medications. It may have been possible for him to have mild or major neurocognitive disorders but his age and sudden onset make it unlikely.

**Figure 3:**
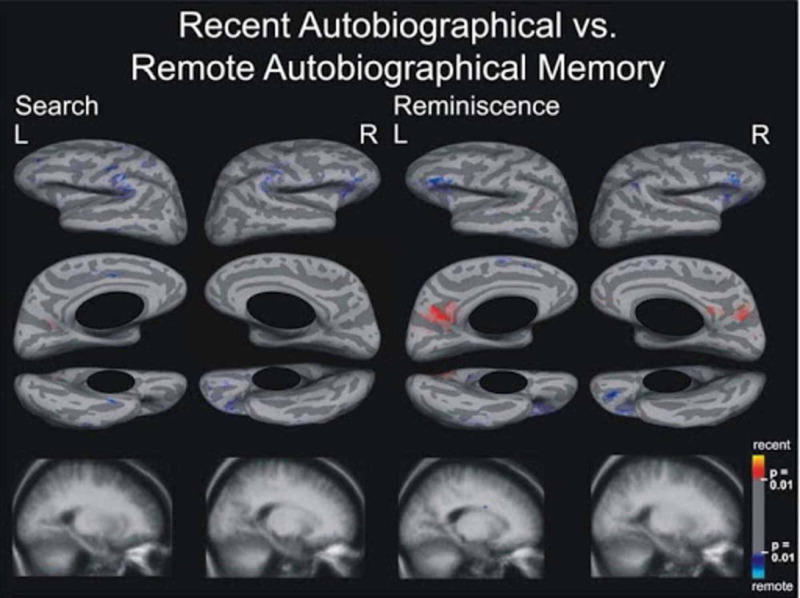
Increased activation noted in bilateral inferior frontal lobes, right hemisphere orbital, and left hemisphere prefrontal areas for remote autobiographical memory [9].

The absence of structural damage to the medial temporal lobe structures in this patient given his loss of remote and recent autobiographical memory is of significant interest. There have been extensive studies on the significance of medial temporal lobe structures in memory formation. In a study by Noulhiane et al. [5], the authors examined the role of the medial temporal lobe structures in autobiographical memory. They compared patients with resections predominantly in the medial temporal lobe (i.e., hippocampus, temporopolar, entorhinal, perirhinal, and parahippocampal cortices) with normal controls. They found that autobiographical memory was impaired in patients with right and left medial temporal lobe resections as compared to normal controls across all time periods. However, there have been studies calling into question the focus on medial temporal lobe structures in autobiographical memory formation. For instance, Kirwan et al. [6] administered an autobiographical interview to three patients with limited hippocampal damage, two patients with large medial temporal lobe lesions, and five controls. The autobiographical interview contained items from remote memory and recent memory. They found that autobiographical recollection was impaired in patients with medial temporal lobe damage when memories were drawn from the recent past, but fully intact when memories were drawn from the remote past. The conclusion from the study is that remote autobiographical memory is likely caused by significant damage outside the medial temporal lobe. In support of this conclusion, Steinvorth et al. [9] reported in a study that areas of functional MRI activation specific to remote autobiographical memory were found only in the inferior frontal lobes bilaterally, the orbital areas in the right hemisphere, and the prefrontal areas in the left hemisphere [9]. The most significant of the findings was that no activation differences appeared in medial temporal lobe structures, even after lowering the statistical threshold [9]. The pattern of activation for remote autobiographical memory reported by Steinvorth et al. is shown in Figure 3 [9]. This challenges the theory of the central role of medial temporal lobes in remote autobiographical memory. Our patient thus could have a loss of autobiographical memory even in the absence of structural damage to the medial temporal lobe structures.

An important consideration is that our patient might have abnormalities in brain activity that are not grossly detectable on CT and MRI. An assessment of brain regions implicated in memory loss may thus need functional MRI or positron electron tomography (PET) studies. The findings from such studies could involve other areas of the brain, or may also involve the medial temporal lobe structures. There is an indication from the literature that such imaging studies may be of benefit in functional or dissociative amnesia [10]. In a study by Brand et al. [10], glucose PET findings in patients with functional or dissociative amnesia suggest right-hemispheric synchronization abnormalities during memory retrieval attempts. It was found that the right frontotemporal region was hypometabolic in a significant number of patients with a significant reduction in the right inferolateral prefrontal cortex [10]. Further studies including PET scans and functional MRI involving all brain regions in patients with autobiographical memory loss may be needed to improve our understanding of the neural correlates of memory formation. Such studies may provide evidence of metabolic dysfunction in otherwise structurally intact brain regions implicated in memory formation, as well as other brain regions not thought to be involved in memory formation.

## Conclusions

Anterograde and retrograde amnesia of autobiographical memory may be present in patients with no structural damage to medial temporal lobe structures. Future large-scale interventional studies are required to find neural correlates of memory functioning. Furthermore, more robust research such as imaging techniques and genetic approaches to find molecular mechanisms and genetic links that underlie memories in both trauma-affected and non-trauma-affected brain areas are warranted.
